# Crystal structure of the sucrose phosphorylase from *Alteromonas mediterranea* shows a loop transition in the active site

**DOI:** 10.1107/S2053230X25004327

**Published:** 2025-06-23

**Authors:** Folmer Fredslund, Marine Goux, Bernard Offmann, Marie Demonceaux, Corinne André-Miral, Ditte Welner, David Teze

**Affiliations:** a DTU Biosustain Technical University of Denmark Kongens Lyngby Hovedstaden Denmark; b US2B, UMR CNRS 6286 University of Nantes 44322Nantes France; Centre for Cellular and Molecular Biology, Hyderabad, India

**Keywords:** sucrose phosphorylases, GH13, marine, loop

## Abstract

Sucrose phosphorylases are essential enzymes regulating sucrose metabolism, and it has been shown that a loop rearrangement is essential to their catalytic cycle. Crystal structures of only six sucrose phosphorylase enzymes are available. Here, we present the crystal structure of a sucrose phosphorylase from a proteobacterium, *Alteromonas mediterranea*, at 2.15 Å resolution. The available sucrose phosphorylase structures have shown that an important conformational change occurs during the catalytic cycle or upon mutagenesis. Interestingly, our data present clear indications of the two major conformations in the same crystal.

## Abstract 2

Sucrose phosphorylases catalyse a bi-bi reaction that interconverts sucrose and phosphate into glucose α-1-phosphate and fructose. Here, we present the first crystal structure of a sucrose phosphorylase from a marine organism.

## 1. Introduction

The energetic metabolism of many organisms requires the interconversion of sucrose and glucose α-1-phosphate (G1P), a reaction catalysed by sucrose phosphorylases (EC 2.4.1.7). These enzymes are carbohydrate-active enzymes, and as such are classified in the CAZy database (https://www.cazy.org; Lombard *et al.*, 2014 ▸) in GH clan H, GH13 family, subfamily 18 (GH13_18; Franceus & Desmet, 2020 ▸). They present a (β/α)_8_-barrel fold, and catalyse the interconversion of sucrose and G1P through two successive pseudo-S_N_2 reactions with a glucosyl-enzyme intermediate. As a result, the products retain the same stereochemistry as the substrates (α-glucose, β-frcutose), and GH13_18 enzymes are thus called retaining enzymes. Despite their central role, until 2019 only a single enzyme structure had been solved, with six having been solved as of 12 November 2024. Few structures of sucrose phosphorylase enzymes have been discussed in scientific publications, such as that from *Bifidobacterium adolescentis* DSM 20083 (*Ba*SP; Mirza *et al.*, 2006 ▸; Febres-Molina *et al.*, 2022 ▸) and that from *Marinobacter adhaerens* (*Ma*GGP; Zhang *et al.*, 2022 ▸). Importantly, the comparison of different structures of *Ba*SP led to the identification of specific loop motions that allow the successive release of fructose and binding of phosphate, modifying the charge content of the active site, particularly with a loop moving by up to 16 Å (Mirza *et al.*, 2006 ▸). Moreover, engineering of this loop has proven to be beneficial to biotechnological applications (Dirks-Hofmeister *et al.*, 2015 ▸; Kraus *et al.*, 2016 ▸). Here, we describe the crystal structure of a sucrose phosphorylase from a marine organism, *Alteromonas mediterranea*, and show that similar loop transitions can be observed in a single crystal of the native enzyme without any substrate.

## 2. Materials and methods

### 2.1. Macromolecule production

The *Am*SP-WT gene (UniProt S5AE64_9ALTE) was ordered from GenScript already cloned in a pET-28b vector with a His_6_-tag in the C-terminal position. *Escherichia coli* BL21(DE3) competent cells (Novagen) were transformed, and clones were selected using LB–agar medium supplemented with 25 µg ml^−1^ kanamycin and confirmed by Sanger sequencing (Eurofins Genomics). Transformed bacteria were grown overnight with shaking at 37°C in 5 ml LB medium supplemented with 25 µg ml^−1^ kanamycin and 0.5%(*w*/*v*) glucose. On the next day, 200 ml LB auto-inducible medium containing 1%(*w*/*v*) glucose and 25 µg ml^−1^ kanamycin was incubated with 2 ml overnight culture. The cells were grown with shaking at 25°C. After 24 h, the cells were centrifuged (ThermoScientific, Sorvall RC6 Plus, rotor SLC 4000, 30 min, 4150*g*, 19°C) and the pellet was resuspended in NPI-5 buffer (50 m*M* NaH_2_PO_4_, 300 m*M* NaCl, 5 m*M* imidazole–HCl pH 8.0; 5 ml per gram of pellet) in the presence of 5 µg ml^−1^ DNAse I, 250 µg ml^−1^ lysozyme and 1 m*M* phenylmethyl­sulfonyl fluoride. Total protein extracts were obtained by sonication. The suspension was centrifuged (ThermoScientific, Sorvall Legend X1R centrifuge, rotor FIS-8x50cy, 20 min, 12 000*g*, 4°C) to remove cell debris. The protein was purified from the supernatant by immobilized metal ion-affinity chromatography (IMAC) using Protino Ni–NTA agarose beads (Macherey-Nagel) equilibrated with NPI-5. After washing with 2 × 10 column volumes (CVs) of NPI-10 buffer (50 m*M* NaH_2_PO_4_, 300 m*M* NaCl, 10 m*M* imidazole–HCl pH 8.0) and 10 CV of NPI-20 buffer (50 m*M* NaH_2_PO_4_, 300 m*M* NaCl, 20 m*M* imidazole–HCl pH 8.0), the purified protein was eluted fivefold with 1 CV of NPI-250/DTT buffer (50 m*M* NaH_2_PO_4_, 300 m*M* NaCl, 250 m*M* imidazole–HCl, 0.5 m*M* DTT pH 8.0). The protein concentration was determined by UV absorbance at 280 nm (NanoDrop 1000, Thermo Scientific) and the purity was confirmed by Coomassie-stained 12% SDS–PAGE. The protein was further purified by size-exclusion chromatography on a HiLoad 16/600 Superdex 200 gel-filtration column (GE Healthcare) equilibrated with 25 m*M* HEPES, 50 m*M* NaCl, 0.5 m*M* DTT pH 7.0. Elution was performed in the same buffer at a flow rate of 0.8 ml min^−1^. Fractions with an OD_280 nm_ of >0.015 at 70 min were pooled and concentrated. Macromolecule-production information is summarized in Table 1 ▸.

### 2.2. Crystallization

**Figure 1:**
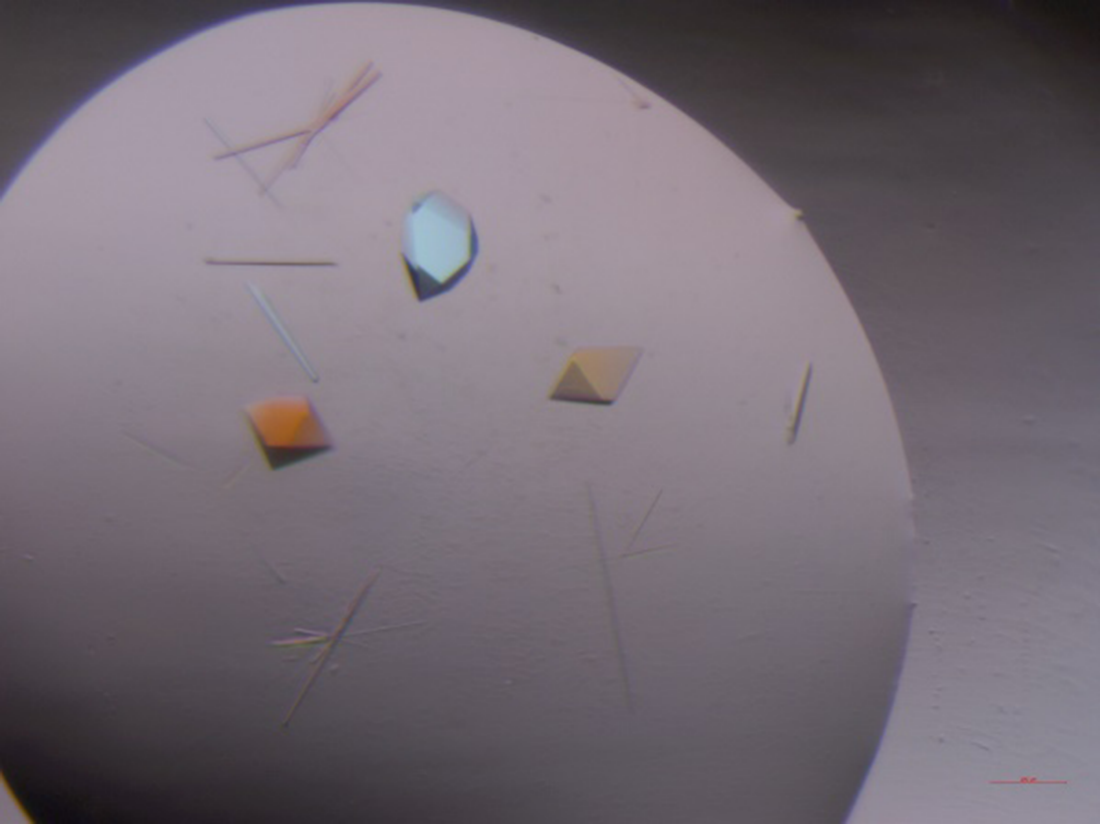
Crystals grown in a SWISSCI MRC 2-Well plate.

*Am*SP at a concentration of 6.9 mg ml^−1^ was set up for crystallization in 96 SWISSCI MRC 2-Well plates using a Crystal Gryphon (Art Robbins Instruments), with the commercial screens SG1 (Hampton Research) and PACT (Jena Bioscience), using 150 or 200 nl protein followed by reservoir to give a total volume of 300 nl. Crystals appeared after three days in several conditions; Fig. 1 ▸ shows the crystals that give rise to the best data set from SG1 condition G5 consisting of 60%(*v*/*v*) Tacsimate (a mixture of titrated organic salts) at pH 7. The crystals were cryoprotected with 20% ethylene glycol before flash-cooling in liquid nitrogen. Crystallization information is summarized in Table 2 ▸.

### 2.3. Data collection and processing

Diffraction data were collected on the MASSIF-3 beamline at the European Synchrotron Radiation Facility (ESRF). A total of 3600 diffraction images were collected with a flux of 1.28 × 10^11^ photons s^−1^ over 21.6 s, corresponding to a dose of 0.11 MGy (Bury *et al.*, 2018 ▸). The output from the automated beamline processing procedure *XDSAPP* was used in refinement after applying a slightly stricter resolution cutoff. Data-collection and processing statistics are summarized in Table 3 ▸.

### 2.4. Structure solution and refinement

The phase problem was solved by molecular replacement. As a model, the sucrose phosphorylase from *B. adolescentis* (*Ba*SP; PDB entry 1r7a; Mirza *et al.*, 2006 ▸) was used after preparation using *Sculptor* (Bunkóczi & Read, 2011 ▸) and a TFZ score of 21.6 was obtained with the *Phaser* software (McCoy *et al.*, 2007 ▸). An initial round of automated model building was followed by several iterations of refinement with *phenix.refine* (Afonine *et al.*, 2012 ▸) and manual model building in *Coot* (Emsley & Cowtan, 2004 ▸). Refinement statistics are summarized in Table 4 ▸.

## 3. Results and discussion

**Figure 2:**
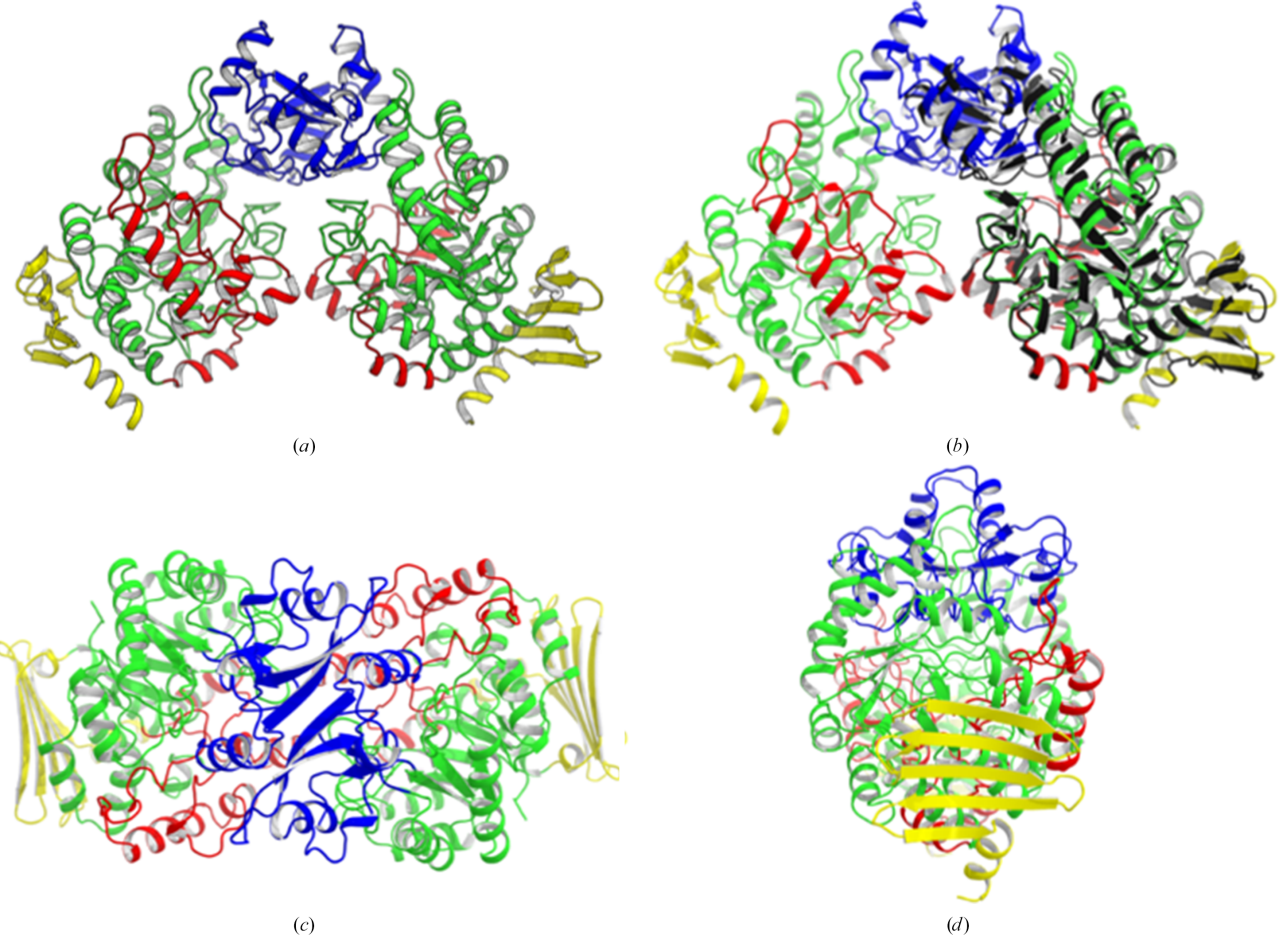
Spatial organization of the domains in the *Am*SP dimer (the catalytic domain A, dimerization domain B and domains Bp and C are represented in green, blue, red and yellow, respectively). (*a*) View of the two molecules in the asymmetric unit. (*b*) Superimposition of the structures of *Am*SP and *Ba*SP (PDB entry 1r7a, black). (*c*, *d*) Rotations of 90° along the *x* and *y* axes, respectively.

*Am*SP appears as a functional dimer, with two molecules (one dimer) in the asymmetric unit. Each monomer is constituted by four domains named A, B, Bp and C. Domain A is the catalytic domain, while the dimer is formed mostly by interactions between the B domains (Fig. 2 ▸), similarly as in *Ba*SP (Mirza *et al.*, 2006 ▸).

**Figure 3:**
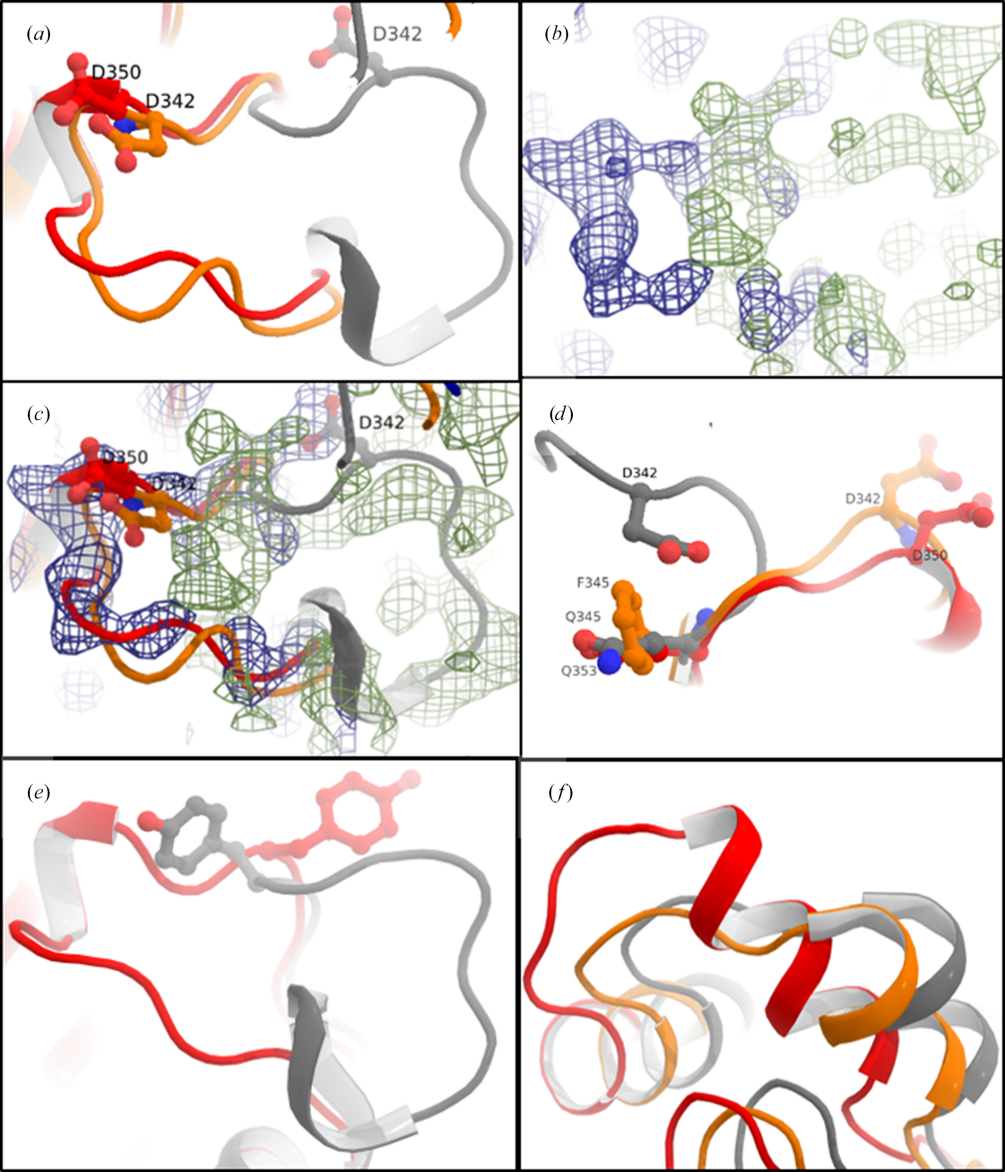
Comparison of loop A in *Am*SP, *Ba*SP and *Ba*SP-Q345F (in red, grey and orange, respectively; corresponding to PDB entries 7znp, 1r7a and 5c8b). (*a*) View of loop A from above. (*b*) The same view as the top left with the electron-density maps of PDB entry 7znp corresponding to the 2*F*_o_ − *F*_c_ map at a 3σ cutoff (blue, representing electron density) and to the *F*_o_ − *F*_c_ difference map at a 3σ cutoff (green, representing disagreement between the model and the electron density). (*c*) The same view with the overlay of the residues and the electron-density maps of PDB entry 7znp. (*d*) View of loop A from its C-terminal side. (*e*) Emphasis on the flip of Tyr352. (*f*) Emphasis on the α-helix which precedes loop B.

An interesting feature in this *Am*SP structure is the position and the flexibility of the alanine-rich loop A. Indeed, both *Ba*SP and *Am*SP present a ATGAAA motif (residues 333–338 and 341–346, respectively) conferring high flexibility to the so-called loop A, which continues up to residues 343 and 351, respectively. It has been reported that for phosphorylation to take place, Asp342 in *Ba*SP (Asp350 in *Am*SP) in loop A moves out of the active site, decreasing the negative charge in the active site and thus reducing electronic repulsion with the incoming phosphate (Mirza *et al.*, 2006 ▸). Indeed, a single negative charge difference can completely preclude a phosphate molecule from binding in an active site, discriminating between hydrolase and phosphorylase activity (Teze *et al.*, 2020 ▸). Interestingly, this loop repositioning can also be induced by engineering, with the mutation Q345F leading to a particularly efficient transglysosylase (PDB entry 5c8d; Kraus *et al.*, 2016 ▸). In the *Am*SP structure, we observe a loop A position that closely matches that of the covalent glucosyl-intermediate of *Ba*SP and that of *Ba*SP-Q345F (Fig. 3 ▸). Thus, despite *Am*SP being an apo enzyme, and not mutated, it seemed to be primed in a configuration favouring phosphorylation. Accordingly, mutants of *Am*SP appear able to catalyse transglycosylation (Goux *et al.*, 2024 ▸). Moreover, the electron density also clearly indicated that the loop conformation typical of an apo, wild-type enzyme was also present in the crystal (Fig. 3 ▸). This highlights that loop A presents two stable conformations with a low energy barrier between them. This low energy barrier is likely due to the flipping of Tyr352 (Fig. 3 ▸*e*). Compared with the *Ba*SP and *Ba*SP-Q345F structures, *Am*SP also presents a slightly more elongated σ-helix (comprising residues 127–136), which directly precedes loop B (Fig. 3 ▸*f*). The motions of loop B have also been shown to be important for the catalytic cycle of sucrose phosphorylases (Mirza *et al.*, 2006 ▸); however, the conformation of loop B in *Am*SP did not seem to differ significantly from that of *Ba*SP.

## Supplementary Material

PDB reference: sucrose phosphorylase from *Alteromonas mediterranea*, 7znp

Raw data for sucrose phosphorylase from Alteromonas mediterranea: https://doi.org/10.15151/ESRF-DC-1114624654

## Figures and Tables

**Table 1:** Macromolecule-production information

Source organism	*Alteromonas mediterranea*
DNA source	Synthetic
Cloning vector	pET-28b
Expression vector	pET-28b
Expression host	*E. coli* BL21(DE3)
Complete amino-acid sequence of the construct produced	MGSIRNGVQLITYADRLGDGNIESLTNLLDGPLKGLFKGVHILPFYYPYDGEDAGFDPIDHTTVDERLGDWNNIKKLGESVDIMADLIVNHMSGQSEAFTDVLKKGRESEYWPLFLTKEDVFSGNDQAEIDEQIAKVFRPRPTPFFSDYEVGIETDSTETVPFWTTFTSNQIDIDVESELGKEYLSSILQSFTESNVDLIRLDAAGYAIKRAGSNCFMLEETFEFIEALSKRARTMGMQCLVEIHSHYQTQIDIAARCDSVYDFALPPLVLHTLFTKDASALAHWLSISPRNCFTVLDTHDGIGIVDVGASGDKPGLISADAINALVEQIHVNSNGESKKATGAAANNVDLYQVNCTYYDALGKDDFAYLVARAIQFFSPGIPQVYYGGLLAAHNDMELLANTNVGRDINRPYLTTAMVEDAIQKPVVKGLMQLITLRNENKAFGGAFDVTYTDNTLVLSWSNDGDAASLTVDFAAMDATINTVSNGEESTLSIGALLAHHHHHH

**Table 2:** Crystallization

Method	Sitting drop
Plate type	SWISSCI MRC 2-Well
Temperature (K)	295
Protein concentration (mg ml^−1^)	6.9
Buffer composition of protein solution	25 m*M* HEPES, 50 m*M* NaCl, 0.5 m*M* DTT pH 7.0
Composition of reservoir solution	60%(*v*/*v*) Tacsimate pH 7.0
Volume and ratio of drop	200 nl protein and 100 nl reservoir solution
Volume of reservoir (µl)	60

**Table 3:** Data collection and processing Values in parentheses are for the outer shell.

Diffraction source	MASSIF-3, ESRF
Wavelength (Å)	0.9677
Temperature (K)	100
Detector	EIGER 4M
Crystal-to-detector distance (mm)	175.39
Rotation range per image (°)	0.1
Total rotation range (°)	360
Exposure time per image (s)	0.006
Space group	*C*222
*a*, *b*, *c* (Å)	132.46, 143.56, 72.80
α, β, γ (°)	90, 90, 90
Mosaicity (°)	0.150
Resolution range (Å)	45–2.15 (2.23–2.15)
Total No. of reflections	521095 (43233)
No. of unique reflections	38027 (3658)
Completeness (%)	99.64 (97.60)
Multiplicity	13.7 (11.8)
〈*I*/σ(*I*)〉†	11.14 (0.68)
*R*_r.i.m._	0.041 (0.703)
Overall *B* factor from Wilson plot (Å^2^)	60.64

† CC_1/2_ was used as the cutoff for the resolution limit, and the mean *I*/σ(*I*) falls below 2.0 at 2.35 Å

**Table 4:** Structure solution and refinement Values in parentheses are for the outer shell.

Resolution range (Å)	42.20–2.15 (2.20–2.15)
Completeness (%)	99.6
σ Cutoff	*F* > 1.33σ(*F*)
No. of reflections, working set	70651 (4515)
No. of reflections, test set	2040 (131)
Final *R*_cryst_	0.185 (0.3652)
Final *R*_free_	0.229 (0.3576)
No. of non-H atoms	
Protein	3896
Ligand	2
Water	107
R.m.s. deviations	
Bond lengths (Å)	0.004
Angles (°)	0.614
Average *B* factors (Å^2^)	
Protein	65.3
Ramachandran plot	
Most favoured (%)	98.2
Allowed (%)	1.8

## Data Availability

The final models and diffraction data have been deposited in the Protein Data Bank (https://www.wwpdb.org/) as PDB entry 7znp and the raw data are available from the ESRF data archive at https://doi.org/10.15151/ESRF-DC-1114624654.
